# How and why patients made Long Covid

**DOI:** 10.1016/j.socscimed.2020.113426

**Published:** 2021-01

**Authors:** Felicity Callard, Elisa Perego

**Affiliations:** a University of Glasgow School of Geographical and Earth Sciences, University of Glasgow, United Kingdom; b University College London Institute of Archaeology, UCL, United Kingdom

**Keywords:** Chronic illness, Citizen science, COVID-19, Long Covid, Long-hauler, Patient activism, Patient groups, SARS-CoV-2

## Abstract

Patients collectively made Long Covid – and cognate term ‘Long-haul Covid’ – in the first months of the pandemic. Patients, many with initially ‘mild’ illness, used various kinds of evidence and advocacy to demonstrate a longer, more complex course of illness than laid out in initial reports from Wuhan. Long Covid has a strong claim to be the first illness created through patients finding one another on Twitter: it moved from patients, through various media, to formal clinical and policy channels in just a few months. This initial mapping of Long Covid – by two patients with this illness – focuses on actors in the UK and USA and demonstrates how patients marshalled epistemic authority. Patient knowledge needs to be incorporated into how COVID-19 is conceptualised, researched, and treated.

## Highlights

• Patients made visible the persistence and heterogeneity of COVID-19 symptoms.
• Patients experiencing long-term symptoms (‘long-haulers’) made Long Covid.
• Long Covid challenges early clinical and governmental assumptions about Covid.
• Knowledge travelled from patients through media to formal health and policy channels.
• Patient expertise and knowledge should be incorporated in the pandemic evidence base.

## Credit author statement

Felicity Callard, Elisa Perego: Conceptualization, Investigation, Writing - original draft preparation, Writing – Reviewing and editing

## 1 Out of collective suffering

Long Covid describes a longer, more complex course of illness than that emerging from initial, formal reports from Wuhan (The Novel Coronavirus Pneumonia Emergency Response Epidemiology Team, 2020; World Health Organization, 2020a). We document how and why patients made Long Covid – and cognate term long-haulers – and why this matters for patient activism and pandemic policy-making. While most patients initially had ‘mild’ COVID-19 and were not hospitalized, many experienced life-threatening symptoms as well as other traumatic events, often without healthcare support. Thousands of patients collectively made visible heterogeneous and complexly unfolding symptoms: most were not commonly acknowledged within many healthcare and policy channels in early pandemic months.

How scientists engage with knowledge about new diseases affects how meaning about a disease accrues, how terminology solidifies, and which evidence is prioritized. In a global health crisis, we need contributions made by those with wide ranges of expertise – including, crucially, patients. There is a long history of those outside formal clinical channels refining or contesting knowledge produced within biomedicine. Mental health survivors have challenged diagnostic categories and developed different ways to conceptualize distress and recovery (Sweeney et al 2009); HIV/AIDS activists have changed how biomedical scientific research is conducted and how drugs are accessed (Robins, 2004); those with long-term/chronic conditions have created patient knowledge and ways of living (Pols, 2013; Kingod, 2018); political organizations have made clear the biopolitical stakes of health and illness (Nelson, 2011). These patient and lay contributions have often been ignored or underacknowledged by conventional actors, which has intensified patient suffering and societal inequalities. We need to learn from these episodes and ensure that patient contributions to the coronavirus pandemic are fully acknowledged and incorporated into policy making .

The speed of publication of COVID-19 research means epistemic authority rapidly consolidates around particular actors. Crucial leads might be lost as background noise – particularly when made by patients, whose expertise is less frequently validated. Long Covid now appears in peer-reviewed publications (e.g. Altmann and Boyton, 2020; Greenhalgh et al., 2020). Many now writing about it are not aware of whence and why it arose. By starting to map Long Covid and related iterations (including ‘long-haulers’) which have started to emerge in the English language, we aim to ensure their patient origins are evidenced within the peer-reviewed literature, as well as emphasize why conventional health actors ought to include these contributions within formal scientific practices.

Long Covid as a patient-made illness carries the potential to change how the pandemic unfolds. It has already challenged dominant assumptions about morbidity and disease severity: as science writer Ed Yong has argued, Covid ‘[l]ong-haulers … are redefining COVID-19’ (Yong, 2020b). There are strong reasons to argue that Long Covid is the first illness to be made through patients finding one another on Twitter and other social media. Both of us have Long Covid and have contributed to making it. Here, we focus on contributions from the UK and USA, while emphasizing the need for research on patient contributions from other countries – including China, South Korea, and Spain, which were affected earlier in the pandemic.

## 2 Exploding ‘mild’ covid

The February WHO-China Report stated ‘the median time from onset to clinical recovery for mild cases is approximately 2 weeks and is 3–6 weeks for patients with severe or critical disease’ (World Health Organization, 2020a). Early reports focused on severe pneumonia; the ‘mild’ was a capacious category including some with pneumonia. Many citizens were told most patients would experience mild illness and rapid recovery. The UK's Chief Medical Officer Chris Whitty stated, in May, ‘the great majority, probably 80%, will have a mild or moderate disease, might be bad enough for them to go to bed for a few days, not bad enough for them to have to go to the doctor’ (Refer video at 46 minutes in 10 Downing Street, 2020).

Emollient descriptions of mild illness did not fit with people's often overwhelming experiences. In March, patients started sharing experiences on social media, drawing attention to possible Covid-related sequelae (e.g. Pope, 2020). In April, newspapers started publishing first-person accounts documenting how challenging recovery from COVID-19 could be (e.g. Lowenstein, 2020). Articles often drew on resources developed within patient-founded groups – e.g. Body Politic COVID-19 Support Group (https://www.wearebodypolitic.com/covid19, founded by Fiona Lowenstein and Sabrina Bleich), and Facebook groups (e.g. https://www.facebook.com/groups/longcovid, founded by Claire Hastie).

On 5 May, the *British Medical Journal* (*BMJ*) published Paul Garner's account of suffering seven weeks through a ‘roller coaster of ill health, extreme emotions and utter exhaustion’ (Garner, 2020). Garner, an infectious diseases professor, also appeared in a feature (Harding, 2020b), which, by 10 August, had been read over 1 million times (Harding, 2020a). Garner's account travelled internationally, gathering a wider patient community around what he termed the Covid ‘long tail’. Garner reported patients took his account to medical appointments to provide evidence of the realness of their symptoms (Colbert et al. n. d.). Patient-made evidence in the form of one case study was important when no peer-reviewed articles had yet documented long-lasting symptoms. Non-hospitalized patients were at that point literally beyond the gaze of biomedical research. Patients also challenged ‘mild’: Felicity Callard, then seven weeks into Covid, anatomized the ‘mild’, emphasizing how little was known about whether mild illness was actually mild (Callard, 2020).

On 11 May, an all-patient team published the first survey of prolonged symptoms reporting over 50 (including cognitive impairment/fatigue, chills/sweats, appetite loss), many of them cyclical (Assaf et al., 2020); the chance of full recovery by day 50 after onset was less than 20% (see also Lambert, 2020). Other patient-led initiatives included Gez Medinger's videos (https://www.youtube.com/channel/UCln_SCEd4JiGkHIUZd1VlXw), and Kate Porter's ‘COVID-19 Recovery Awareness’ (https://www.c19recoveryawareness.com).

## 3 Long-haulers

In early June, Yong published ‘COVID-19 can last for several months’ (Yong, 2020a). Yong's reputation and the article's calibre ensured wide circulation. (By 19 August, 1 million visitors had read the online version [personal communication, Yong, August 19, 2020]). Yong's article featured nine people, described support groups ‘hosting thousands’, and noted some called themselves ‘long-haulers’. ‘Long-haulers’ came from Amy Watson, patient convener of ‘Long haul Covid fighters’, who derived it from a trucker hat she was wearing when getting a test (Porter, 2020). ‘Long-haulers’ soon moved into general circulation.

## 4 Prevalence

Accounts by patients, support groups, surveys, and digital advocacy (e.g. the LongCovidSOS film https://www.longcovidsos.org/film) brought vivid case studies to wide audiences, expanded knowledge of symptoms, and made demands. But patient-led efforts had to contend with epistemological gaps. One concerned prevalence: how common were ‘long-haulers’? Patients made strategic use of any incidence data available. Notable here was the app-run COVID-19 Symptom Study (King's College London, 2020), the only large study releasing data regarding Covid symptoms and duration. In May, we learned that ‘about one in 20 Covid patients experience long-term on-off symptoms’ (Harding, 2020b). In June, the study reported ‘one in ten people may still have symptoms after three weeks, and some may suffer for months’ (COVID-19 Symptom Study App, 2020). Patients made incidence data into hashtags (#Covid1in20 – then #Covid1in10). Not until 9 July did an article confirm a high incidence of long-term symptoms, showing 87.4% of hospitalized patients reported at least one symptom 60 days after onset (Carfi et al., 2020).

## 5 Long Covid

Long Covid as a term gained consistency in just a few weeks. #LongCovid was first used by Elisa Perego, from Lombardy (a very hard-hit, early hotspot) on 20 May (Perego, 2020), as a contraction of long-term Covid illness, to summarize her experience of disease as cyclical, progressive, and multiphasic. She used #LongCovid to intervene ontologically in formulations of COVID-19 in peer-reviewed papers – by complicating the ‘biphasic’ disease pathway common in peer-reviewed publications (e.g., Lescure et al., 2020), and pointing to multiple sequelae. In June, #LongCovid became increasingly prominent – complementing other hashtags used by emergent patient collectivities (e.g. #apresJ20 in French, #covidpersistente in Spanish; #MitCoronaLeben in German; #koronaoire in Finnish; #長期微熱組 in Japanese; see also patient websites https://www.apresj20.fr and https://apuakoronaan.fi).

‘Long Covid’ moved from social to print media in late June when a newspaper described how doctor Jake Suett had joined a ‘Long Covid’ support group (Keay, 2020). This group (https://www.longcovid.org) changed its name to ‘Long Covid Support Group’ in response to growing use of #LongCovid – having previously added #Covid1in20 to its name on 23 May in response to the COVID-19 Symptom Study incidence data (personal communication, Claire Hastie, August 19, 2020).

On 7 July, a BBC interviewer asked Suett about ‘this Long Covid, as they call it’ (BBC News 24, 2020a), and the Royal College of General Practitioners noted general practice was ‘preparing for an “influx” of patients with ‘long Covid’ (Royal College of General Practitioners, 2020). On 8 July, *New Statesman* published a piece by a doctor with ‘#LongCovid’ in the standfirst: the hashtag indicated the term's emergence through social media (Whitaker, 2020). On 10 July, *BMJ* published a blog by medical doctors with ‘chronic COVID-19 symptoms’ titled ‘Patients’ experiences of “longcovid” are missing from the NHS narrative’ (Lokugamage et al., 2020). On 14 July, *BMJ* published the clinical feature ‘What do we know about “long Covid”?’ (Mahase, 2020). Two days later, UK MP Andrew Gwynne asked in Parliament about support for long-term Covid patients, identifying himself as being in ‘Week 17 of long Covid viral fatigue’ (HC Vol 678, 2020). On 17 July, Long Covid reached a journal: an immunological article described ‘the chronic aftermath of infection posed by chronic, so-called “long-COVID” cases’ (Altmann and Boyton, 2020).

Then qualifiers such as inverted commas started to disappear. A BBC interview on 20 July noted: ‘We have … been talking more and more about long Covid’ (BBC News 24, 2020b). On 26 July, UK MP Layla Moran noted the All-Party Parliamentary Group (APPG) on Coronavirus was hearing from many suffering from ‘long-lasting effects of coronavirus, they call it long-Covid’ (Sky News, 2020). Ten days later, when the APPG gathered oral evidence, MPs used Long Covid straightforwardly (All-Party Parliamentary Group on Coronavirus, 2020).

By mid-August Long Covid had stabilised into a recognisable ‘scientific object’ (Daston, 2000) – even as its precise contours remain subject to debate. On 21 August, the Director-General of the WHO met advocates from across the world (Sacks, 2020), after Maria Van Kerkhove (WHO COVID-19 Technical Lead) contacted the Long Covid SOS group in July to discuss advocates' demands (World Health Organization, 2020c). Long Covid and Long-haul Covid were used in communications within and around the event. On 8 September, Matt Hancock, UK Secretary of State for Health and Social Care, used Long Covid when speaking to a parliamentary committee (Parliamentlivetv, 2020).

## 6 Uptake within formal scientific channels

Acknowledgement of prolonged Covid illness by the wider scientific community often occurred subsequent to patient efforts. In May, Van Kerkhove responded to a question about ‘people suffering from symptoms for many weeks’ stating, ‘Thus far there is very little evidence to suggest there are people who are persistently suffering from COVID-19’ (World Health Organization, 2020b). Only on 19 June did Van Kerkove note that some ‘mild patients’ were experiencing ‘some lingering effects’ (World Health Organization, 2020d). She recognised the need to ‘understand what recovery looks like’, mentioning ‘our collaborations with clinicians and medical professionals’ (World Health Organization, 2020d).

Anthony Fauci, the Director of the US's National Institute of Allergy and Infectious Diseases, and an internationally prominent pandemic advisor, did not initially invoke clinical networks in relation to long-term symptoms but rather – notably – referred to ‘anecdotal’ patient data. Here, patient-made material from informal channels was used as evidence prior to data being formally available from scientific studies. On 9 July, Fauci stated ‘if you look anecdotally, there is no question that there are a considerable number of individuals who have a post-viral syndrome’. Fauci mentioned as evidence ‘chat groups that you just click on and see people who recovered that really do not get back to normal’ (Fox, 2020). In late July, Avindra Nath and Jeanne Billioux (US National Institutes of Health) wrote on ‘long-haul Covid’ (Nath and B Jeanne Billioux, 2020), picking out autonomic symptoms. Which evidence they drew on for their typology was unclear; a broader range of symptoms had already been reported in patient-led surveys.

In July, peer-reviewed studies started reporting sequelae (cardiological, neurological) even in ‘mild’ COVID-19 (Paterson et al., 2020; Puntmann et al., 2020; Tenforde, 2020). These papers circulated widely online under the Long Covid hashtag. Patients continued to initiate medical discussions on Twitter – speculating about aetiologies and treatments. By late July, scientists on Twitter used ‘Long Covid’ themselves (e.g. on 28 July, Eric Topol launched a challenge to ‘demystify … what accounts for #LongCovid’ (Topol, 2020)). Patients were not always explicitly included as addressees in such discussions.

## 7 An unstable term

The aetiology of Long Covid is currently under scrutiny and may be multiple (Perego et al., 2020). Definitions of COVID-19 itself remain unstable: the pathology has been defined as a respiratory, cardiovascular, endothelial, or systemic condition (Lescure et al., 2020; Marini and Gattinoni, 2020). Whether COVID-19 will, for some, survive viral persistence and develop into a disease-specific, chronic or permanent condition, and/or whether SARS-COV-2 infection generates a new autoimmune disease remains unknown (Topol et al., 2020). Many patients, through use of Long Covid, wish to keep aetiological possibilities open while much remains unclear.

Nonetheless, Long Covid has been under pressure to conform to or be subsumed within other terms. Some already exist (e.g. post-viral fatigue syndrome, ME/CFS). Others are COVID-19 specific (e.g. ‘post-Covid syndrome’ (BBC, 2020)). Some in the ME/CFS community have suggested some Long Covid cases might morph into ME/CFS (Shepherd, 2020), and there are already lively exchanges both over the relationship between Long Covid and ME/CFS, over forms of solidarity between two patient communities experiencing ‘chronic viral-induced illness’, and diagnostic terminology for persistent symptoms. It is crucial, we argue, that Long Covid patients with different disease experiences and pathways are included in deliberations over terminologies used for long-term symptoms/illness: the history of medicine demonstrates much is at stake in terms of how diseases are modelled and understood (Dumit, 2006).

## 8 Ethics and exploitation

Patients continue to experience epistemic injustice (Fricker, 2011) – their long-term symptoms misunderstood, or reduced to anxiety (Yong, 2020b); patients of colour and those with disabilities are particularly at risk. Many struggle to receive care, particularly if they were not tested. The ownership and interpretation of patient-held materials pose ethical and practical challenges. Huge datasets of symptoms and potential therapeutics are held in informal patient archives, comprising significant evidence from first cohorts. Comparably rich information might not be accrued by research studies fast enough to help emerging Covid hotspots and direct policy. Urgent attention needs to be given to ensuring ethical means of engaging with these data: it is essential these acknowledge the forms of solidarity that underpinned their emergence while learning from previous episodes of patient data exploitation (Prainsack, 2017).

## 9 Conclusion

Long Covid challenges common assumptions that were in place in the early pandemic and which often persisted despite patient testimony. In the making of Long Covid, conventional hierarchies of evidence, and normative routes for scientific dissemination were frequently disrupted. A patient-led survey released on a collective's website; the self-appellation of a community after a trucker hat; a single case study authored by a patient, and taken by others to clinical appointments; the circulation of a hashtag first used by a patient to refine the model of COVID-19 in published articles. Through such acts Long Covid solidified, moving from patients to multiple new actors – with some conventional scientific actors arriving on the scene after patients. We note the speed of consolidation: Long Covid and Long-haul Covid moved from patients to meetings with the WHO in just a few months. Will Long Covid persist as a term, or will this ‘scientific object’, which, by late August 2020, achieved solidity, break up into other classifications (Callard, 2020)?

In HIV/AIDS science, ‘knowledge emerge[d] out of credibility struggles’ involving activists who challenged divisions between scientist ‘insiders’ and lay ‘outsiders’ (Epstein, 1996). As with AIDS, Covid patients have used various tactics to claim epistemic authority – often by marshalling authority relating to professional expertise (e.g. Medinger is a film director, Lowenstein a writer). It was frequently patients with professionally validated forms of authority who were more able to access formal routes, which helped stabilize patient-made terms (e.g. medical doctors [e.g. Suett on BBC], academics in medicine/health [e.g. Garner in *BMJ* blog], parliamentarians [e.g. Gwynne]). Many clinicians and researchers at the forefront of Long Covid research and advocacy (e.g. Alwan, 2020a, Alwan, 2020b; Lokugamage et al., 2020) have had Covid. Others carved out authority by dint of being patients (e.g. Hastie presented at APPG). Those who introduced the terms ‘long-haulers’ and ‘Long Covid’, as well as many of those providing labour in COVID-19 support and advocacy, are women. How authority has been recognised intersects in overdetermined ways with gender, ethnicity, class, and disability. Discrimination – including racism, sexism, ableism – helps explain why patients from marginalized/minoritized communities, many of whom are central to making Long Covid, have been denied platforms, and sometimes have decided not to place themselves in the spotlight to discuss a disease that compounds discrimination. Some with professional power, e.g. Yong, amplified patient contributions. Others have not.

Long Covid has a strong claim to be considered the first illness to be collectively made by patients finding one another through Twitter and other social media. This phenomenon opens multiple questions: How does Long Covid shift assumptions about the role of media in the production of science? Are Long Covid patients developing different ways of authorising their knowledge claims from those used elsewhere? How might the specific characteristics of COVID-19 and Long Covid (particularly the apparent heterogeneity of illness pathways and the current difficulty in knowing who will develop protracted illness/disability) matter in conceptualising ‘acute’ and ‘chronic’ illnesses (see Manderson and Wahlberg, 2020)? Do responses to Long Covid patients from conventional scientists differ from those experienced by others with protracted illness (e.g. the tendency to describe ME/CFS advocacy as ‘militant’ (Blease and Geraghty, 2018), and if so why?

Crucially, researchers' accounts of patient activism during the pandemic need to learn from other epidemics: privileging white men in narratives about AIDS occluded contributions by and impacts on those in the Global South (Cheng et al., 2020). Our short communication on Long Covid is one small piece of a much bigger story. We have explained how and why patients in, or with links to, certain English-speaking countries collectively produced knowledge, circulated it through multiple media, and broadened what counts as evidence. We end by calling for the work of more actors to be documented, and for patients’ ongoing contributions to be recognised and used to combat the suffering of multitudes.

## Funding

10.13039/100010269Wellcome Trust (209513/Z/17/Z).
